# A robust method to isolate *Drosophila* fat body nuclei for transcriptomic analysis

**DOI:** 10.1080/19336934.2021.1978776

**Published:** 2021-10-06

**Authors:** Vanika Gupta, Brian P. Lazzaro

**Affiliations:** a Department of Entomology, Cornell University, Ithaca, NY, USA; b Cornell Institute of Host-Microbe Interactions and Disease, Cornell University, Ithaca, NY, USA

**Keywords:** Fat body, single-cell sequencing, scSeq, RNAseq, transcriptome, profiling, metabolism, immune response

## Abstract

Gene expression profiles are typically described at the level of the tissue or, often in *Drosophila*, at the level of the whole organism. Collapsing the gene expression of entire tissues into single measures averages over potentially important heterogeneity among the cells that make up that tissue. The advent of single-cell RNA-sequencing technology (sc-RNAseq) allows transcriptomic evaluation of the individual cells that make up a tissue. However, sc-RNAseq requires a high-quality suspension of viable cells or nuclei, and cell dissociation methods that yield healthy cells and nuclei are still lacking for many important tissues. The insect fat body is a polyfunctional tissue responsible for diverse physiological processes and therefore is an important target for sc-RNAseq. The *Drosophila* adult fat body consists of fragile cells that are difficult to dissociate while maintaining cell viability. As an alternative, we developed a method to isolate single fat body nuclei for RNA-seq. Our isolation method is largely free of mitochondrial contamination and yields higher capture of transcripts per nucleus compared to other nuclei preparation methods. Our method works well for single-cell nuclei sequencing and can potentially be implemented for bulk RNA-seq.

## Introduction

The insect fat body is a highly multifunctional tissue that regulates diverse physiological processes, such as nutrient storage and metabolic control, immune responses to infection, and production of proteins essential for egg provisioning [1]. This single tissue thus shares function with several vertebrate tissues, including liver and adipose tissue. The fat body is an extremely dynamic tissue that exhibits dramatic expression changes in response to physiological stimulus [2]. It, therefore, is an important tissue to understand. The diverse functions of the fat body imply that there may be cellular heterogeneity within the tissue, and spatially restricted morphological and functional heterogeneity have previously been observed [3,4]. Single-cell RNA-seq (sc-RNAseq) is a technique that enables transcriptomic profiling of individual cells [5], which could be invaluable for studying the fat body. However, the success of sc-RNAseq relies on clean, gentle, and rapid dissection of the tissue of interest and dissociation of individual cells. The adult fat body of *Drosophila melanogaster* is a large and fragile tissue that is distributed throughout the body [1] and is, therefore, more difficult than other tissues to analyse at a single-cell level. In this manuscript, we compare four methods for isolating fat body cells and nuclei prior to sc-RNAseq. In our hands, isolation of intact fat body cells is infeasibly challenging and results in unacceptably high cellular mortality (see Supplement). Standard protocols to isolate individual nuclei using a sucrose gradient or low-speed centrifugation were successful in capturing nuclei but carried unacceptably high levels of contamination with mitochondria (see Supplement). We ultimately developed a modified method that combines careful tissue dissection and fixation, cell lysis, and nuclear isolation over a sucrose density gradient to generate high-yield, high-purity nuclear isolations that are suitable for transcriptomic profiling. Our method resulted in high-quality RNA that was suitable for single-cell transcriptomic profiling by RNA sequencing [6].

## Results

**Figure 1.**
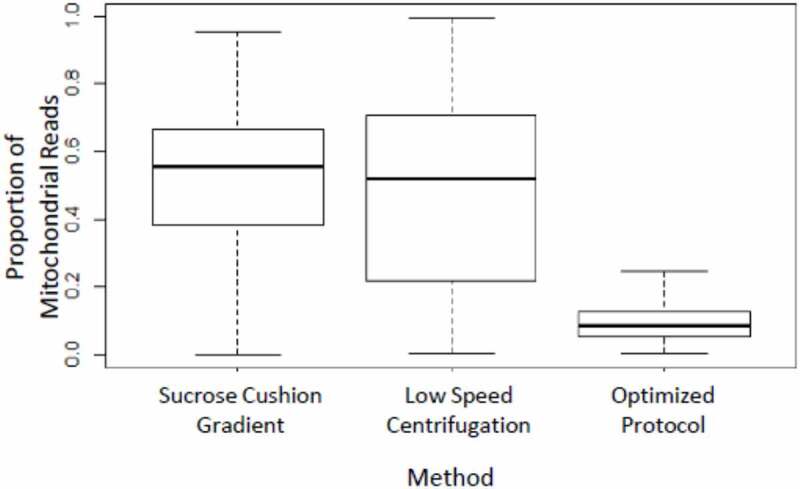
Box plot showing the proportion of mitochondrial reads after three different nuclei preparation protocols. Our optimized protocol yields the lowest proportion of mitochondrial reads

Enzymatic dissociation is the most common method to dissociate a tissue into a single cell suspension. We noticed that the *Drosophila* fat body cells are fragile and rapidly die when subjected to enzymatic dissociation (described in Supplement). Therefore, this was not a suitable approach from our perspective. Studies [7–9] have shown that transcriptomic profiles correlate strongly between nuclei and cells, meaning that nascent transcripts in the nucleus are broadly representative of the standing mRNA pool in the cell and suggesting they could be used for transcriptomic profiling. We tested various methods to prepare nuclei suspension from the fat body tissue. We tested two methods, sucrose cushion gradient and low-speed centrifugation (described in Supplement), which resulted in an unacceptably high proportion of mitochondrial reads (Figure 1) in the sequencing data. Our objective was to generate high-quality nuclear gene expression data with low contamination from mitochondria and other cellular debris, so we optimized a protocol to isolate individual intact nuclei.

**Figure 2.**
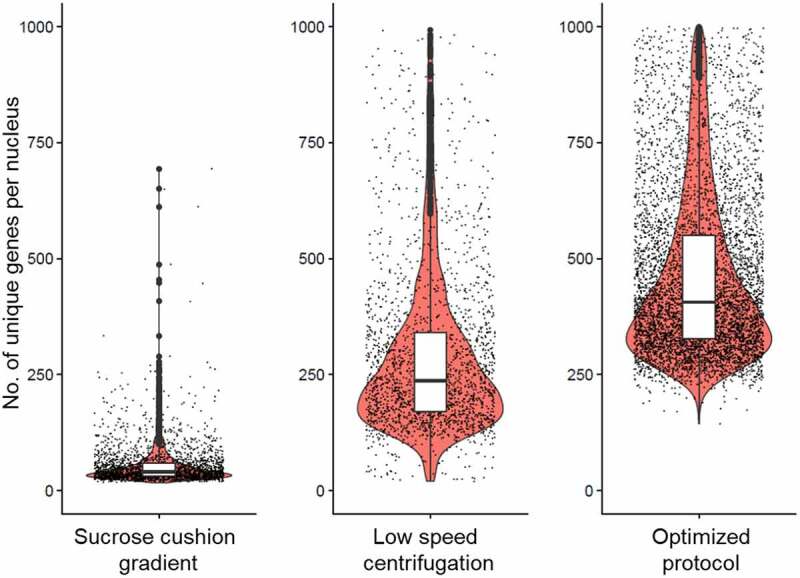
Box-overlaid-violin plot showing the number of unique expressed genes detected per nucleus after isolation with three different nuclei preparation protocols. The expressed gene counts were generated from the single-nucleus sequence data analysed using Seurat [10]. Our optimized protocol yielded a higher number of unique expressed genes detected per nucleus

Using our optimized protocol, we saw a dramatic reduction in mitochondrial contamination. Fewer than 5% of the purified nuclei were associated with 20% or higher mitochondrial reads (Figure 1), and there was a considerable improvement in the number of reads and distinct genes obtained per nucleus using the optimized protocol (Table 1). As a part of routine sample quality control for single-cell data analysis, we only consider nuclei expressing 20 genes or more when considering genes that are expressed in at least five cells. Upon analysing the raw dataset with Seurat v3.1 [10], our optimized protocol showed a significantly higher number of genes captured per nucleus (Figure 2). The dramatic reduction in the number of mitochondrial reads and a larger number of unique expressed genes detected per nucleus in our optimized protocol represents a major improvement over the other two protocols tested. Our method was robust and gave a similar value for low mitochondrial read contamination and a high number of genes captured when applied to eight fat body samples subjected to different biological treatments [6]. The optimized protocol we developed is as follows:

**Table 1.** Summary of genes detected in nuclei prepared using three different methods

	Sucrose cushion gradient	Low-speed centrifugation	Optimized protocol
Median expressed genes per nucleus	42	73	443
Total expressed genes across all nuclei	6,923	9,339	10,125

### Reagents required

#### Adult haemolymph-like saline (HLS)

For dissection and storing tissues, haemolymph-like saline of osmolarity suitable for adult flies [11] was made using the recipe provided by Cold Spring Harbour Laboratories (CSHL, http://cshprotocols.cshlp.org/content/2013/11/pdb.rec079459.full). HLS was filter-sterilized, aliquoted and stored at 4°C until needed^12^

#### Adult haemolymph-like saline (CSHL)

2 mM CaCl_2_ (Sigma-Aldrich# C5426)

5 mM KCl (Sigma-Aldrich# P9541)

5 mM HEPES (pH = 7.4) (Fisher Scientific# AAJ16924AE)

8.2 mM MgCl_2_ (Sigma-Aldrich# M8266)

108 mM NaCl (VWR# BDH8014)

4 mM NaHCO_3_ (Sigma-Aldrich# S5761)

1 mM NaH_2_PO_4_ (Sigma-Aldrich# S9638)

10 mM Sucrose (Sigma-Aldrich# S9703)

5 mM Trehalose (Sigma-Aldrich# T0167)

Adjust the pH to 7.5. Filter sterilize and store in aliquots at 4°C

#### Fixation buffer

4 parts Methanol (Sigma-Aldrich# 179957) + 1-part HLS (Pre-chilled at −20°C)

#### Hypotonic isolation buffer (HIB)

10 mM HEPES (pH = 7.4)

10 mM KCl

2.5 mM MgCl_2_

0.5 mM Spermidine (Sigma-Aldrich# S2626)

0.15 mM Spermine (Sigma-Aldrich# S3256)

0.02% Digitonin (Sigma-Aldrich# D141)

#### Hypotonic sucrose buffer (HSB)

10 mM HEPES (pH = 7.4)

10 mM KCl

2.5 mM MgCl_2_

0.01% NP40

0.3 M Sucrose

#### Wash and suspension buffer (WSB)

1X phosphate-buffered saline (PBS, pH = 7.4)

2% BSA (Sigma B4287)

0.2 U/µl RNaseIN (Roche# 3335399001)

#### Protocol

1. Autoclave micro-centrifuge tubes (Eppendorf# 022431048), pipette tips, Dounce homogenizers (Wheaton# 357538, 357542).
2. Keep micro-centrifuge tubes and Dounce homogenizers at 4°C so that everything is chilled before use.
3. Anaesthetize adult flies using CO_2_. Pick a fly using fine forceps (Fine Science Tools# 91150–20) and submerge the fly in cold HLS. With the fly submerged in HLS, use the fine forceps and pull at the posterior tip of the abdomen. With the help of spring scissors (Fine Science Tools# 15000–00), incise laterally along the cuticle. Open the cuticle and carefully remove the ovaries, gut, and Malpighian tubules, exposing the fat body layer attached to the cuticle.
4. Transfer the dissected fat body tissue, still attached to the cuticle, to Fixation Buffer. Incubate on dry ice for 5–10 minutes.
5. Transfer the dissected tissue to 1 mL Dounce homogenizer (Wheaton# 357538) containing 1 mL HLS on ice.
6. Repeat steps 3–5 to pool the desired number of dissected fat bodies. We pooled 40 tissues (Figure 1). *Note*: 40 abdomens reliably yield approximately 10^6^ nuclei as counted by Trypan blue staining using Bio-Rad automated cell counter (TC20).
7. Carefully remove the HLS from the tube, leaving enough liquid such that tissues do not dry out.
8. Add 1 mL of ice-cold HIB. Allow tissues to swell up in hypotonic buffer for 5 minutes.
9. Dounce the tissues gently twice with a loose pestle and once with a tight pestle. Keep the pestle submerged in the buffer during lysis, avoiding bubble formation.
10. Transfer tissues along with hypotonic isolation buffer to 7 mL Dounce homogenizer with a wide-mouth P-1000 pipet tip (VWR# 89049–160). Wash the 1 mL homogenizer with 1 mL of HIB and transfer it to a 7 mL homogenizer. Add another 2 mL of HIB and Dounce twice with a tight pestle.
11. Transfer lysate to 5 mL centrifuge tube. Pass lysate through a 22 G syringe (BD# 309631) 3–5 times carefully. Do not allow the abdominal cuticle to pass through the syringe.
12. Transfer supernatant to 2 mL LoBind® centrifuge tubes (Eppendorf# 022431048) and spin at 800 g for 7 minutes @ 4°C.
13. Carefully remove and discard the supernatant without disturbing the pellet.
14. Gently resuspend pellet in HSB and centrifuge at 1500 g for 10 minutes @ 4°C.
15. Carefully remove supernatant and resuspend the pellet in 500 µl of WSB.

We further removed cellular debris using sucrose gradient centrifugation. Using Sigma kit (NUC201-KT), we followed the protocol using instructions provided by 10X Chromium [12]. The protocol is as follows:

1. Prepare Sucrose Cushion Buffer I (SCB I) by mixing 2.7 ml Nuclei PURE 2 M sucrose cushion solution (Component NUC201-KT) and 300 µl Nuclei PURE sucrose cushion buffer (Component NUC201-KT). Keep it on ice.
2. Transfer 500 µl of SCB I to a 2.0 ml LoBind® microcentrifuge tube. Keep it on ice.
3. Take 900 µl of SCB I and add it to 500 µl of nuclei suspension from Step 15 in the protocol. Mix thoroughly but gently.
4. Carefully overlay the nuclear suspension mixed with SCB I (from Step 18) onto the 500 µl SCB I in the LoBind® microcentrifuge tube (from Step 17). Do not mix the two layers.
5. Without disturbing the layers, transfer the LoBind® tube to a microcentrifuge pre-chilled at 4°C.
6. Centrifuge samples at 3500 g for 20 minutes at 4°C.
7. Carefully remove the tube from the centrifuge and place it on ice.
8. Remove 1950 µl of the supernatant, leaving 50 µl in the tube. If the nuclei pellet is not visible, then leave around 100–150 µl of supernatant.
9. Resuspend the pellet in WSB to a total volume of 500 µl.
10. Pass the sample through a Flowmi® cell strainer (BAH136800040) with 40 µM pore size. The sample may need to be passed twice if there is excess debris.
11. Immediately proceed to the sequencing protocol or any other downstream use of the purified nuclei.

## Discussion

Here we provide an optimized method for preparing a nuclear suspension suitable for transcriptomics. Our method uses a combination of detergents and gentle centrifugations over a sucrose gradient to generate a purified nuclear suspension with low mitochondrial contamination and a higher number of genes captured per nucleus. Fixing the samples immediately upon dissection preserves the quality of the RNA and results in excellent transcriptomic data [6]. Detection of a greater number of expressed genes per cell allows researchers to make more accurate predictions about the biology of single cells. The ability to robustly capture more genes per cell has driven advancement in single-cell transcriptomics protocols, such as Smart-seq protocols [13] or different chemistries of 10x genomics 3ʹ sequencing kits [14].

Single-cell sequencing technology requires dissociation protocols that can provide a high yield of intact living cells from which a standing mRNA pool can be reliably recovered. However, cell dissociation protocols are delicate, and the precise protocol needs to be specifically optimized for the tissue type of interest. This may prove challenging for fragile tissues, such as the insect adult fat body, whose cells are unstable and rapidly die upon dissociation. On the contrary, nuclei can be isolated from any cell or tissue type. Since the nascent transcript pool in the nucleus is strongly correlated with the standing mRNA pool in the cytoplasm [7–9], sequencing nuclei can provide a reliable alternate strategy for sequencing any tissue at single-cell resolution.

Although our protocol was specifically developed for *Drosophila melanogaster*, we expect that it can be broadly applied. The fat body is a major tissue in all insects and performs several key functions including immune response, metabolism, production of egg yolk and vitellogenins, and xenobiotic detoxification. Single-cell/nucleus sequencing provides a tremendous opportunity to study this crucial insect tissue and understand the cellular basis for functional diversity within the tissue. We expect that the protocol we present here can be readily applied to the fat body of non-*Drosophila* insects and can be adapted for other tissue types as well.

## Supplementary Material

Supplemental Material
