# Supplementary material for: A robust method to isolate Drosophila fat body nuclei for transcriptomic analysis
Source: Fly (Austin). 2021 Oct 6;16(1):62–7. doi: 10.1080/19336934.2021.1978776 (PMC8500699; doi:10.1080/19336934.2021.1978776)
Supplement: Supplemental Material [file KFLY_A_1978776_SM7446.pdf]

**A robust method to isolate *Drosophila* fat body nuclei for transcriptomic analysis**

## Supplementary text

Before optimizing the nuclei isolation method described in the main text, we tested three methods to prepare cell or nuclei suspension from the fat body tissue. These methods worked sub-optimally for us, resulting in either low cellular viability or high mitochondrial content. Conditions that we tested are as follows:

**1. Enzymatic dissociation:** Several protocols[1] use papain, collagenase, trypsin or a combination of enzymes to digest tissue and liberate individual cells. We tested several enzymes, including papain, collagenases, trypsin, TrypLE, and Liberase™, to dissociate the fly fat body tissues into single cells in varying enzyme concentrations and incubation duration. We estimated the percentage of viable cells in the suspension upon dissociation using a Trypan blue exclusion assay and automated Bio-Rad cell counter (TC20). Generally, all enzymatic dissociation methods that we tested resulted in a rapid fat body cell death. Lowering the incubation temperature slows the enzyme activity[2] and can lead to lower cell death. However, even when we incubated our tissues at 4°C with trypsin for 6 hours, we recovered only 15-20% viable cells.

We attempted to use fluorescence-assisted cell sorting (FACS) to recover viable fat body cells after enzymatic dissociation. To label the fat body tissue, we used the Gal4-UAS system to drive the expression of EGFP in the adult fat body tissue. We expressed EGFP under the control of the c564-GAL4 driver, which is strongly expressed in the fat body but can also be expressed in hemocytes, oenocytes, salivary glands and male reproductive tissues such as accessory glands, seminal vesicles (Bloomington Stock Center #6982) with UAS-EGFP to drive the expression in the fat body. After brief enzymatic dissociation (15 minutes) with collagenase I, we used FACS to sort EGFP+ cells from non-fluorescing cells followed by another sort using DAPI live-dead staining to separate live, intact cells from dead cells and debris. We observed variable EGFP expression of EGFP+ cells with no distinct bimodal distribution separating EGFP+ cells from EGFP- cells (Figure S1). Additionally, the sorting revealed a large amount of cell debris and DAPI+ nuclei, suggesting cell death upon dissociation. These results confirmed that the fat body tissue is extremely fragile, making it almost impossible to use enzymatic dissociation to recover individual viable cells. Therefore, irrespective of the driver, dissociating fat body cells into viable cells remain a challenge for downstream single-cell transcriptomic application. We also tested the c564-GAL4 driver controlling the expression of UAS-mCherry.NLS (Bloomington Stock Center #38424) as a label to drive fluorescence in fat body nuclei. In this case, we found 16N ploidy to be the most abundant nucleus population recovered, compared to 2N and 4N in the control sample (Figure S2). This change in ploidy profile could have consequences for the biology of the tissue. While other expression drivers might yield greater tissue specificity of expression, the fragility of the fat body tissue would remain an issue for any method based on sorting and variable ploidy associated with the genotypes expressing different labels could remain a concern.

**2. Sucrose cushion gradient centrifugation for nuclei preparation:** In this method, tissue homogenate prepared in the hypotonic buffer is passed over a sucrose gradient and centrifuged. Cell debris and other organelles are trapped at specific sucrose densities. In contrast, the lighter nuclei form a fraction at the bottom of the gradient. We prepared a fat body homogenate using a Dounce homogenizer and hypotonic buffer. The homogenate was centrifuged at 500g for 5 minutes. The pellet was resuspended in PBS containing 2% BSA, and the suspension was then passed through a sucrose gradient following the 10X Chromium sucrose cushion protocol[3] where

samples were centrifuged at 13,000g at 4°C. However, our sequencing results showed about 50% mitochondrial reads in our samples, making it unsuitable for our transcriptomic experiments (Figure 1).

3. **Low-speed centrifugation for nuclei preparation:** Low-speed centrifugation in hypotonic buffers is typically used[3] to remove cell debris and mitochondria and pellet nuclei. We centrifuged fly homogenate at 500g for 15 minutes and carefully removed the supernatant. The nuclear pellet was washed three times with hypotonic buffer containing 1U/µl RNase inhibitor and centrifuged at 800g for 15 minutes at 4°C. The pellet was resuspended in PBS containing 2% BSA. Following this protocol, our sequencing results still showed unacceptably high contamination with mitochondrial reads. Only 25% of the read sets from individual isolated nuclei contained less than 20% mitochondrial reads (Figure 1). The number of median genes obtained per cell was 73 compared to 443 in our optimized protocol described below (Table 1).

**Supplementary Figures**

Figure S1

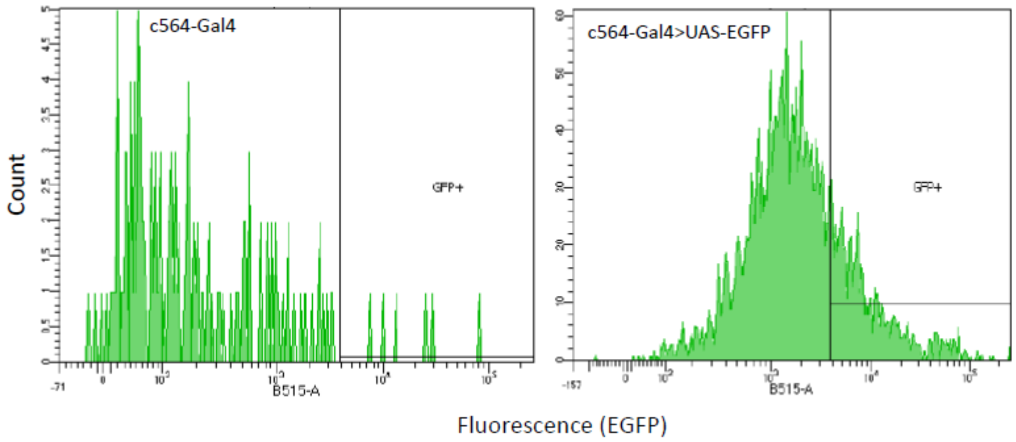


Figure S1: Sorting EGFP+ cells from EGFP- cells shows variable EGFP expression and a lack of separate peak of c564-EGFP+ cells, reflecting few intact viable cells.

Figure S2

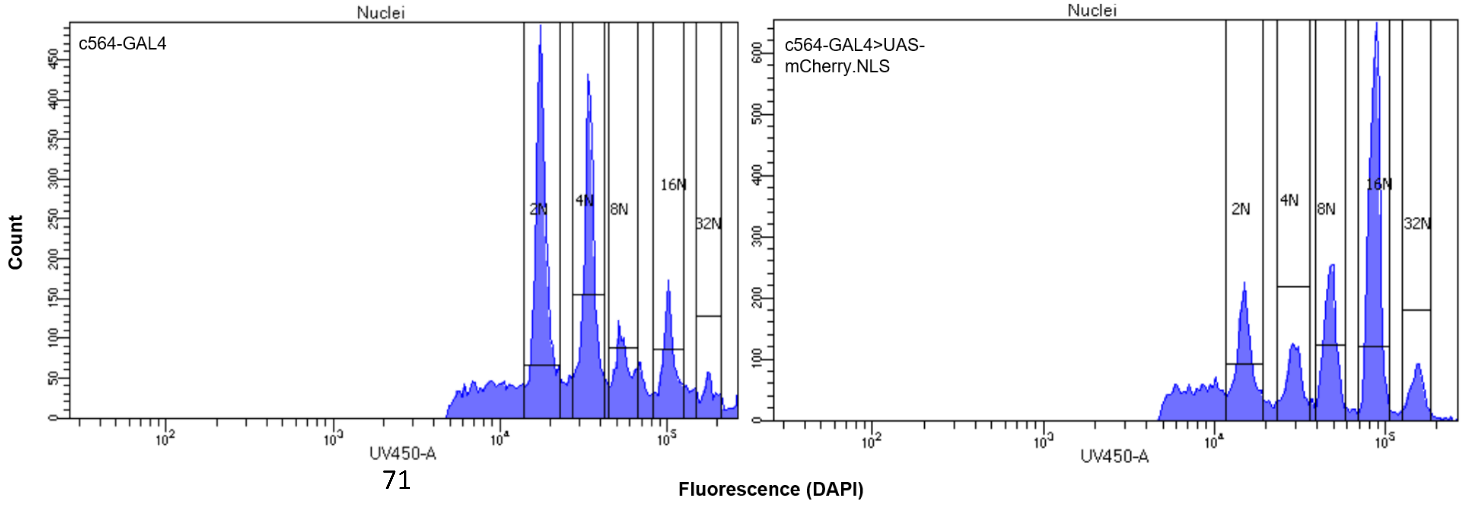


Figure S2: Histogram of DNA content of DAPI+ nuclei show differences in ploidy in control (c564-GAL) and flies expressing mCherry.NLS+ from the c564-GAL4 driver (c564-GAL4>UAS-mCherry.NLS).

## References


1. Li H. Single-cell RNA sequencing in Drosophila: Technologies and applications. Wiley Interdiscip Rev Dev Biol 2020; :1–16.
2. Reichard A, Asosingh K. Best Practices for Preparing a Single Cell Suspension from Solid Tissues for Flow Cytometry. Cytom Part A 2019; 95:219–26.
3. 10X Genomics. Sample Preparation Demonstrated Protocols Isolation of Nuclei for Single Cell RNA Sequencing. 2017;
